# Effective lung-targeted RNAi in mice with peptide-based delivery of nucleic acid

**DOI:** 10.1038/s41598-019-56455-2

**Published:** 2019-12-27

**Authors:** Kaido Kurrikoff, Krista Freimann, Kadi-Liis Veiman, Elin Madli Peets, Andres Piirsoo, Ülo Langel

**Affiliations:** 1 Institute of Technology, University of Tartu, Nooruse 1, 50411 Tartu, Estonia; 2 Department Biochemistry and Biophysics, Stockholm University, S. Arrheniusv 16B, SE-106 91 Stockholm, Sweden

**Keywords:** Chemical modification, Gene delivery, Chemical modification, Drug delivery, Nucleic acids, Peptides

## Abstract

We have previously developed efficient peptide-based nucleic acid delivery vectors PF14 and NF55, where we have shown that these vectors preferentially transfect lung tissue upon systemic administration with the nucleic acid. In the current work, we have explored the utilization and potential of these vectors for the lung-targeted gene therapy. Accordingly, we assessed the efficacy of these peptides in (i) two different lung disease models – acute lung inflammation and asthma in mice and (ii) using two different nucleic acid cargos – siRNA and pDNA encoding shRNA. Using RNAi against cytokine TNFα, we showed efficient anti-inflammatory effects in both disease models and observed decreased disease symptoms. Our results highlight the potential of our transfection vectors for lung gene therapy.

## Introduction

RNAi and nucleic acid biomolecules represent huge potential for the future medicine. The single major obstacle in utilization of RNAi in gene therapy is the problem of the “delivery”^1^ – it is notoriously difficult to get the nucleic acid molecules into the target cell cytoplasm or nucleus. Chemical transfection methods aim to solve a part of this problem by vectorizing nucleic acid into condensed form, whereupon it is easier to protect the cargo against degradation and translocate through the cell membrane.

As an example of efficient transfection methods, we have previously developed two cell penetrating peptide (CPP) vectors that, in addition to cell cultures, demonstrate efficient *in vivo* activity^2,3^: i.e. they are able to deliver plasmid DNA (pDNA) or siRNA upon systemic injection^4^. These peptide-like entities are NF55^2^ and PF14^3^; they have an N-terminal fatty acid component that drives the formation of micelles in aqueous environment and they are extremely efficient in condensing the nucleic acid into nanoparticles^2^ (Table 1).

**Table 1:** The sequences of the peptides PF14 and NF55.

Name	Sequence
PF14	Stearyl- AGYLLGKLLOOLAAAALOOLL-NH_2_
NF55	(Stearyl- AGYLLG)δ-OINLKALAALAKAIL-NH_2_

Another part of the nucleic acid delivery problem is that even if efficient transfection is achieved, the therapeutic should be specifically targeted to the diseased tissue. Nanoparticle-encapsulated drug targeting seems to be a challenging problem, because by far the most frequently used targeting method has been utilizing the passive accumulation/enrichment of the nanoparticle into the tumor tissue^5–7^. Although there are numerous reports that utilize various targeting strategies^8^, a number of conflicting requirements to the whole delivery system^4,9^ make it difficult to achieve efficient delivery.

In the current work we took a different approach and instead of trying to adjust the drug delivery method to the disease requirements, we asked ourselves – which disease could benefit from our particular drug delivery system? As we show in this report, the drug carrier does not distribute evenly throughout the organism and some conditions could be predisposed to better treatment outcome than the others. Accordingly, as detailed subsequently, we took advantage of the native liability of our peptide carriers to transfect lungs and focus on treatment potential of the pulmonary pathologies.

## Results and Discussion

### Peptide vectors PF14 and NF55 offer native lung transfection

**Figure 1:**
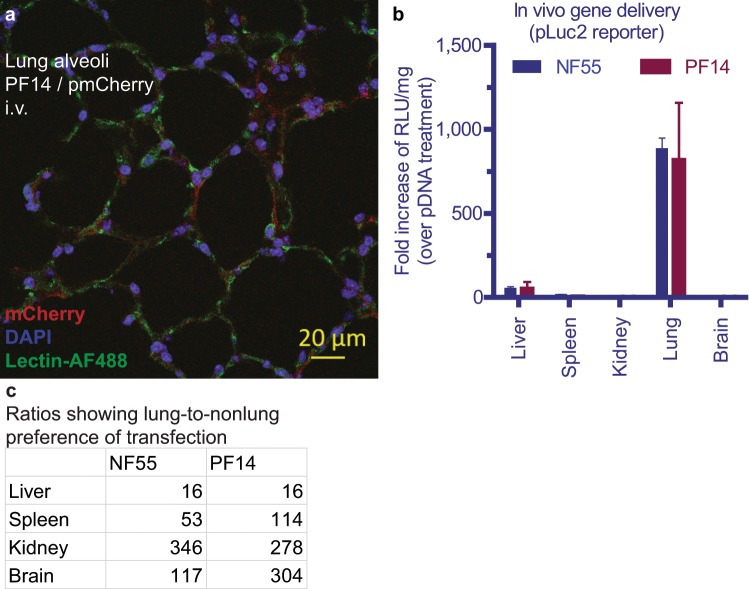
Lung transfection using peptide vectors PF14 and NF55. Transfection of the lung with a reporter plasmid encoding (**a**) mCherry, (**b**) Luciferase. (**c**) Lung-to-nonlung transfection ratios according to the (**b**) demonstrate strong lung preference.

When we look at the nucleic acid transfection after systemic administration of the peptides PF14 and NF55 (Table 1), there is strong transfection in the lung (Figs. 1a and S1) and a clear preference of the signal towards lungs (Fig. 1b), although the physical accumulation of the cargo nucleic acid occurs mainly in liver (Supplementary Fig. S2). Obviously, from the gene therapeutic point of view it is relevant to look at the transfection, rather than physical accumulation. The lung-to-nonlung transfection ratio is 16…350-fold enrichment among the main organs Fig. 1c. Comparing the two organs with highest transfection, i.e. lung vs liver, 16-fold lung enrichment can be observed. Therefore, we hypothesized that the peptide carriers exert huge potential for lung-targeted delivery and it is reasonable to assess their efficacy in pulmonary conditions.

### The lung delivery is safe

It is paramount that the delivery system be safe. A well-known fact from previous studies is that cationic transfection reagents can exert adverse respiratory effects^10–12^. In our previous studies, we have specifically addressed this problem and we recently proposed an alternative “safe” formulation for our peptides and nucleic acid cargo that offers nontoxic transfection^10^. Indeed, we asked a pathologist to assess histological slices from the animals that received the peptides and confirmed that there are no harmful effects (Supplementary Fig. S3) and it is safe to proceed with *in vivo* models.

### RNAi with the peptide vectors reduces LPS-induced lung inflammation

**Figure 2:**
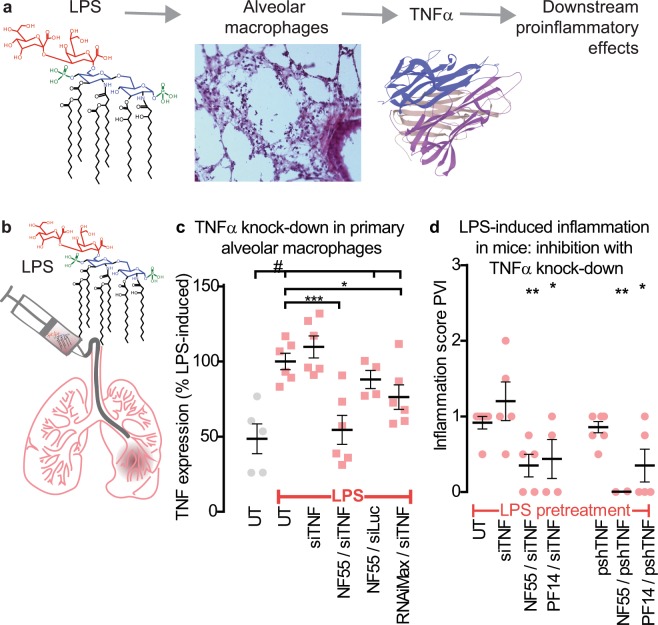
siTNF, delivered by PF14 or NF55 inhibits LPS-induced inflammation. (**a**) Schema illustrating that LPS is a strong inducer of TNFα, which is an early step in subsequent inflammatory cascade. Our strategy is to inhibit inflammation by suppressing TNFα. (**b**) Administration of LPS into the lungs induces acute inflammation. (**c**) Knockdown of TNFα in LPS-activated primary macrophages. 100% represents the average TNFα level of the LPS-induced macrophages. Stars represent post-hoc comparisons after one-way ANOVA (F(5, 27) = 9.4, p < 0.001); #represents comparisons between the UT (no LPS) and the LPS-induced groups of UT, siTNF, NF55/siLuc, RNAiMax/siTNF, where the p-values ranged between 0.05 and 0.001. siLuc is a control siRNA with irrelevant target (luciferase). (**d**) Inhibition of LPS-induced inflammation in mice by knocking down TNFα in the lungs either with siTNF or pshTNF. Inflammation is quantified through patho-histological assessment of perivascular infiltration (PVI). ANOVA F(18, 96.652) = 1.9, p < 0.05; statistical differences represent group comparisons against naked (nonvectorized) siTNF or pshTNF.

Although reporter assays are excellent for the quantification, they contain artificial (albeit well-optimized) biological systems that may not occur in real life. To assess if our gene therapeutic intervention would be achievable in the real biological system, we started with a simple model of lipopolysaccharide (LPS) -induced inflammation. LPS induces acute inflammation that resembles acute and strong bacterial infection. LPS is recognized by macrophages that get activated and start producing and secreting proinflammatory cytokine TNFα, which in turn act as chemotaxis to numerous downstream cytokines and activation signal to other cells (Fig. 2a)^13^. Therefore, inhibition of TNFα represents an early step in the inflammatory cascade and is therapeutically important target.

Because we aimed at inhibiting TNFα with RNAi, we first validated the siTNF in primary macrophages (Fig. 2a). For that, we cultured primary macrophages from the mouse bronchoalveolar lavage, activated these with LPS, and transfected siTNF using RNAiMax or our peptides (Fig. 2b). We could easily knock down TNFα, more efficiently than with RNAiMax (Fig. 2c). This encouraged us to proceed *in vivo* and, using LPS to induce acute inflammation, assess the same inhibition. We induced lung inflammation in mice (Fig. 2b) and again attempted to inhibit TNFα. This time we used both siRNA and a plasmid encoding short hairpin RNA (pshTNF, see Supplementary Fig. S4 and the “Methods” section about construction of the plasmid), administered using our peptides i.v. Using a plasmid offers the theoretical advantage of longer exposure to the therapeutic, because the plasmid constitutively expresses shRNA and the therapeutic is thus naturally “renewed”.

Now the important question was – could the treatment affect inflammation? Since the progression of acute inflammation is extremely fast, and it is not possible to capture first the upregulation and then subsequent knockdown of the TNFα (we could not detect changes in TNFα expression within our 28 h timeframe between LPS administration, siRNA treatment, and tissue collection), we proceeded directly to evaluation of the clinical phenotype. This was assessed from lung histology (Supplementary Fig. S5) and the estimation of the presence of various inflammation markers, such as perivascular, peribronchial, alveolar infiltration of lymphocytes, and edema (Supplementary Fig. S6). Perivascular infiltration scores (PVI, presence of lymphocytes around the vasculature) are shown in Fig. 2d. We were encouraged to see that both of our peptides – NF55 and PF14 – were efficient in reducing lung inflammation and both nucleic acid types –siRNA and plasmid – were efficient (Fig. 2d).

### RNAi with the peptide vectors reduces inflammation in a model of asthma

**Figure 3:**
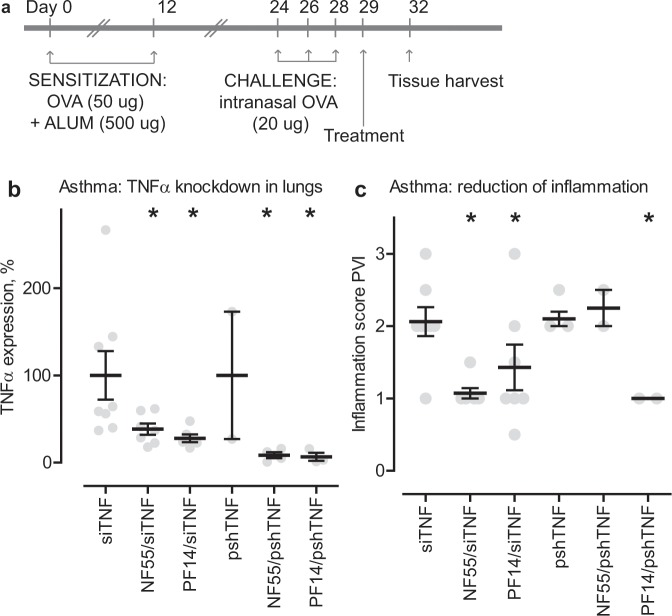
Nucleic acid therapy with siTNF inhibits asthma. (**a**) Schema and experimental plan of OVA/ALUM-induced asthma model, treatment with the test compounds and tissue analysis time points. Asthma was induced by sensitizing the animals with foreign protein (ovalbumin) and an adjuvant (aluminum hydroxide). The treatment consisted of systemic RNAi against TNFα, accomplished via our peptide carrier vectors PF14 and NF55. Treatment efficacy was estimated from qPCR and pathological assessment of the lung tissues. (**b**) Knockdown of TNFα in the lungs of asthmatic mice, qPCR. 100% represent treatments with either naked, nonvectorized siTNF or pshTNF. Stars represent post-hoc comparisons after one-way ANOVA F(5, 24) = 3.5, p < 0.05. (**c**) Inhibition of asthma in mice by knocking down TNFα in the lungs either with siTNF or pshTNF. Inflammation is quantified through patho-histological assessment of perivascular infiltration (PVI). Stars represent post-hoc comparisons after one-way ANOVA F(5, 24) = 4.3, p < 0.01, statistical differences represent group comparisons against naked (nonvectorized) siTNF or pshTNF.

As a final step, we chose a mouse model that allowed treatment and assessment over a longer time period and in chronic pathology – a situation where application of gene therapy is extremely potential. We used a well characterized model of murine asthma, induced by general sensitization with a foreign protein and an adjuvant and then local administration of the sensitizer into the lungs^14^. This was followed by treatment with the siRNA or pDNA (Fig. 3a). With this model, there is a clear and distinct period where inflammatory processes develop (Fig. 3a, days 24–29) and treatment effects take place (days 29–32). Indeed, following the murine asthma model and performing RNAi treatment, qPCR analysis revealed potent knockdown of the TNFα gene, mediated by both siTNF and pshTNF, and with both of our peptides NF55 and PF14 (Fig. 3b). As can be seen from the figure, the untreated animals (or rather, animals receiving naked, nonvectorized nucleic acid) display TNFα gene expression levels ranging from low to very high (up to 300% from the average), whereas the treated animals show mostly low TNFα expression levels with narrow variability. This demonstrates significant gene knockdown in the lung and is in good agreement with our hypothesis about lung targeting with our peptides. Finally, similarly to the LPS model, we assessed the presence of inflammation from the lung histology, following the treatment with nucleic acid therapeutics. As can be seen from the Fig. 3c, Supplementary Figs. S7 and S8, the treatment was effective in most cases: siTNF, vectorized with either of the peptides, as well as PF14 with the pshTNF significantly reduced the PVI score. When comparing the gene knockdown (Fig. 3b) and respective inflammatory phenotype (Fig. 3c), one can observe ubiquitous efficiency of both peptides, with both therapeutic cargoes (siRNA and pDNA) in knocking down the target gene. The reason why quite not the same efficacy is not translated to anti-inflammatory phenotype (reduced inflammation in 3 groups out of 4) may be that that in case of asthma, knockdown of just one gene may not be enough to reverse the whole syndrome, but rather partly inhibits the inflammatory processes. Moreover, it can not be ruled out that other gene targets might have had even stronger therapeutic effect than TNFα.

## Conclusions

In the current report we aimed to demonstrate effective nucleic acid delivery into the lung. We have recently developed two peptides that are well usable *in vivo*^2,3^, because they show excellent efficacy and safety profile. However, their relevance beyond sensitive reporter models and utility in medicine has been demonstrated only in limited cases. As an example of this, we have previously demonstrated cancer delivery by adding targeting components to the delivery vector^3,15^. In the current work however, we utilized the native biodistribution bias of the carrier itself. Indeed, by taking advantage of the underlying property of our CPPs PF14 and NF55 to preferentially and efficiently transfect the lung^2,3^, we just adjusted the vector into preclinical models associated with pulmonary function. Our main aim was to demonstrate the delivery prowess of the peptide vectors and achieve measurable gene modulation in physiological conditions. Indeed, as we hypothesized, the peptide vectors PF14 and NF55 demonstrated efficient gene knockdown through RNAi in lungs, both by using siRNA and plasmid DNA as the cargo therapeutic. Moreover, we also demonstrated measurable physiological changes and achieved beneficial therapeutic effects in preclinical murine models. The current work represents a large step towards clinical application of nucleic acid therapies, using peptide-based drug delivery vectors.

## Methods

All methods were carried out in accordance with relevant guidelines and regulations.

The following software was used to generate the graphical content:

Figure 1A - Zen software (Zeiss); Figs. 1B, 2C,D, 3B,C, S2, S6, S8 - GraphPad Prism; Fig. 2a,b – Symyx Draw, Adobe Illustrator; Figs. 2a,b, 3a - Adobe Illustrator; Fig. S4 – Benchling data export.

### Synthesis of peptides

The peptides (Table 1) were synthesized in a stepwise manner in a 0.1 mmol scale on an automated peptide synthesizer (Biotage Initiator + Alstra) by using fluorenylmethyloxycarbonyl (Fmoc) solid phase peptide synthesis strategy with Rink-amide ChemMatrix resin (0.45 mmol/g loading) to obtain C-terminally amidated peptides as described previously^3^. Briefly, N-terminally stearylated peptides were prepared by treatment of peptidyl resin with 5 equiv of stearic acid (Sigma-Aldrich), 4 equiv of HOBt/HBTU (MultiSynTech), and 8 equiv of DIEA (Sigma-Aldrich) in DMF/DCM for 18 h. The final cleavage was performed by treating with 95% TFA/2.5% TIS/2.5% water for 2 h at room temperature. Peptides were purified by RP-HPLC using a C4 column (Phenomenex Jupiter C4, 5 μm, 300 Å, 250 × 10 mm) and 5–80% acetonitrile (0.1% TFA) gradient. The molecular weight was verified by MALDI-TOF mass-spectrometry (Brucker Microflex LT/SH, USA), and purities were >90% as determined by analytical HPLC.

### Complex formation

Complexes of nucleic acid and the CPP carriers were formed in MQ water, at N/P = 2 (nitrogen/phosphate ratio 2), as detailed in^10^. Briefly, the complexes were formed in 100 µL of MQ, allowed to incubate for 40 minutes, after which 100 µL of 10% glucose was added and immediately injected (final injection in 200 µL in 5% glucose). The standard injected nucleic acid dose was 1.0 mg/kg.

The siRNA sequences are from the ref. ^16^ as follows: siTNFsense 5′-pGUCUCAGCCUCUUCUCAUUCCUGct-3′, siTNFantisense 5′-AGCAGGAAUGAGAAGAGGCUGAGACAU-3′, where the RNA bases are uppercase, DNA bases are lowercase, and “p” is a 5′-phosphate. The oligonucleotides were ordered from Metabion, Germany.

### Development of plasmid encoding the short hairpin (pshTNF)

The plasmid encoding short hairpin respective to the siTNF was constructed into the backbone of the pCpGfree-siRNA (Invivogen pCpGfree-siRNA, cat no kcpgf-sirna). The choice of the backbone was based on the following characteristics: (i) The backbone has been specifically designed for obtaining plasmid devoid of CpG dinucleotides for increased compatibility with the eukaryotes. (ii) Instead of the usual use of CMV or SC40 promoters, the plasmid incorporates mammalian 7SK promoter, again allowing increased mammalian compatibility.

The insert is as follows:

5′-ACCTC-**GTCTCAGCCTCTTCTCATTCCTG-**TCAAGAG-**CAGGAATGAGAAGAGGCTGAGAC-**TT-3′

The siRNA stems are marked with bold. The plasmid map can be found in Supplementary Fig. S4.

### Animal procedures

All the animal experiments and procedures described in this report have been approved by the Estonian laboratory animal ethics committee (the “Project Authorization Committee”, enforced from the Article 36 of the EU Directive 2010/63) with the approval no 110, dated Jun 12, 2017. This committee is certified to evaluate and authorize animal experimentation projects in the EU state and follows the national (Estonian) guidelines (the Animal Protection Act, issued by the Estonian Parliament and enforced with the § 82 of the Act) and EU guidelines (2010/63/EU). Balb/c mice were used, males and females at equal numbers, with the age of 8–10 week old at the time of injecting the test substances (peptide carriers).

### Anesthesia

Standard anesthesia dose is defined as a mixture of 75 mg/kg ketamine (Bioketan, Vetoquinol, France) and 1 mg/kg dexmedetomidine (Dorbene, Laboratorios SYVA, Spain) i.p in saline. Some procedures were performed with reduced amount of anesthetic and in these cases the reduced amount has been indicated in the relevant section. Where relevant, the anesthesia was blocked using the α2-adrenergic antagonist atipamezole hydrochloride (Antisedan, Pfizer), 1 mg/kg s.c (these cases are again indicated in the relevant sections).

### BAL procedure and cultivation of the macrophages

Mice were euthanized immediately prior to lavage by anesthetic overdose. Trachea was exposed and cannulated. 0.5 mL of ice-cold PBS–EDTA (sterile Mg- and Ca-free PBS + 0.6 mM EDTA) was injected into the lungs and using a 3-way valve, aspirated into another syringe. The cycle was repeated with new PBS for 8 times (8 washes with 1 mL of PBS–EDTA). Viable and non-viable cells were counted from the lavage using trypan blue. The lavage was then centrifuged at 450 g × 10 min and the cell pellet was re-suspended in warm RPMI and maintained at standard mammalian cell culture conditions (37 °C, 5% CO_2_).

### LPS-induced acute lung inflammation

In order to induce the inflammation, LPS (2.5 mg/kg in saline) was administered intranasally twice, at 0 h and +24 h. For this, the animals were anesthetized with ½ of the standard anesthetic dose. LPS was delivered by administering 50 uL of the solution dropwise with a regular 200 uL pipette and allowing the animal to inhale the drops. Anesthesia was blocked immediately after the intranasal administration. *In vivo* transfection with siTNFα was performed at +1 h. Lungs were harvested at +28 h.

### Mouse model of asthma

The asthma model was performed as described in^14^, the schema illustrating the experimental plan is in Fig. 3a. The first step is sensitization to ovalbumin (OVA). This is done by injection of 50 μg of OVA (Thermo) adsorbed to 500 μg aluminum hydroxide (Alum, Thermo) in 200 μL PBS, administered on days 0 and 12 i.p. Secondly, the mice are challenged to OVA with 20 μg of intranasal (i.n.) OVA in 50 μL PBS on days 24, 26, and 28. This procedure is performed under ½ dose of anesthesia and anesthesia was blocked after the i.n. administration. The treatment with siRNA or shRNA commenced on day 29 and tissues were harvested on day 32.

### Lung inflation with cryomatrix

For the histological evaluation, the lungs were inflated *postmortem* with 1:1 cryomatrix:PBS (O.C.T embedding matrix, Kaltek, Italy) via intratracheal cannula, removed from the chest cavity, and frozen in 2-methylbutane bath on dry ice. Fresh cryosections (10 μm thick) were fixed with formalin and stained with H&E. As an input to the qPCR, 10–15 lung cryosections (each 15 μm thick) were pooled into a frozen 1.5 mL Eppendorf tube, whereas the heart was discarded.

### Histology and pathological analysis

The tissue sections were assessed by a pathologist in blind manner, i.e. the pathologist received the samples without prior knowledge about the group or treatment. The presence of inflammation in the lungs was scored in semi-quantitative way by assigning a score in the scale of 0 (normal tissue/no inflammation), 1 (mild inflammation/infiltration of the leukocytes), 2 (moderate inflammation), or 3 (severe inflammation), in 0.5-point grades. The pathologist scored the presence of perivascular infiltration (PVI), peribronchial infiltration (PBI), alveolar inflammation (AI), and edema.

### *In vivo* transfection

The transfection complexes (CPP/siRNA or CPP/pDNA) were prepared as described above, in 200 μL per injection, and injected into the tail vein. The reporter gene expression levels were evaluated from the whole tissue homogenate *post mortem* 24 h after single injection as described in detail in^10^.

### qPCR

The tissues were homogenized using Precellys 24-Dual homogenization system (Bertin Technologies, France) with TRIzol reagent (Thermo Fisher Scientific). Before RNA extraction, additional centrifuge step was utilized to reduce the amount of cryomatrix in the sample. TRIzol reagent was added directly onto the frozen cryosections (as per manufacturer’s protocol, 1 mL of TRIzol per 50–100 μg of tissue sample) and thoroughly vortexed to minimize section clumping. The samples were then incubated for 3 min at room temperature, vortexed and centrifuged 16 000 × g for 1 min. Sedimented insoluble fibrous tissue was discarded and supernatant containing the TRIzol-solubilised nucleic acid and protein was used to proceed with normal TRIzol manufacturer’s RNA extraction protocol.

RNA quality was assessed and concentration measured using 1.8% agarose gel and NanoDrop2000, respectively. cDNA was synthesized using SuperScript IV Reverse Transcriptase (Thermo Fisher Scientific) according to the manufacturer’s protocol. qPCR was performed with the SYBR green system, using HOT FIREPol EvaGreen qPCR Supermix (Solis Biodyne, Estonia) according to the manufacturer’s protocol. The obtained Ct were analyzed using 2^−ΔΔCT^ method.

Primers for the qPCR are as follows: Mus musculus TNFα forward: 5′-TACTGAACTTCGGGGTGATTGGTCC-3′, reverse: 5′-CAGCCTTGTCCCTTGAAGAGAACC-3′ and Mus musculus Actin beta forward: 5′-CCACACCCGCCACCAGTTCG-3′, reverse: 5′-TACAGCCCGGGGAGCATCGT-3′. The oligonucleotides were ordered from Metabion, Germany.

### Statistical analysis

The data variability in the figures is described via standard error (s.e.m.). Statistical differences between experimental factors were calculated with the help of a statistical package Statistica (Dell) using ANOVA and individual group comparisons with Fisher LSD *post-hoc*. The significance thresholds are highlighted in the figures as follows: *p < 0.05, **p < 0.01, ***p < 0.001.

## Supplementary information

Supplementary information

## Data Availability

The raw data required to reproduce these findings are available to download from: https://data.mendeley.com/datasets/v8dd56w6wc/draft?a = f43d6782-b98e-40a9-b87c-975aefd1602a
