# Supplementary material for: Effective lung-targeted RNAi in mice with peptide-based delivery of nucleic acid
Source: Sci Rep. 2019 Dec 27;9:19926. doi: 10.1038/s41598-019-56455-2 (PMC6934651; doi:10.1038/s41598-019-56455-2)
Supplement: Supplementary file 1 — Supplementary information [file 41598_2019_56455_MOESM1_ESM.pdf]

Supplementary Information

Effective lung-targeted RNAi in mice with peptide-based delivery of nucleic acid

Kaido Kurrikoff, Krista Freimann, Kadi-Liis Veiman, Elin Madli Peets, Andres Piirsoo, Ülo Langel

Corresponding author Kaido Kurrikoff

Email: kaido.kurrikoff@ut.ee

This Supplementary Information includes:

Figs. S1 to S8

Fig S1. Transfection of Lungs and Liver with NF55 / pmCherry

Fig S2. Biodistribution of pDNA after formulation with PF14 at 24h after i.v. injection

Fig S3. H&E from normal UT mouse and from the animals who were injected with PF14 or NF55 in complex with pDNA

Fig S4. The map of the pshTNF plasmid

Fig S5. Lung transfection with siTNF or pshTNF inhibits LPS-induced inflammation: H&E lung sections

Fig S6. The effect of knocking down TNF in LPS-induced inflammation: the pathology scores

Fig S7. Lung transfection with siTNF or pshTNF inhibits asthma: H&E lung sections

Fig S8. The effect of knocking down TNF in OVA/ALUM-induced asthma: the pathology scores

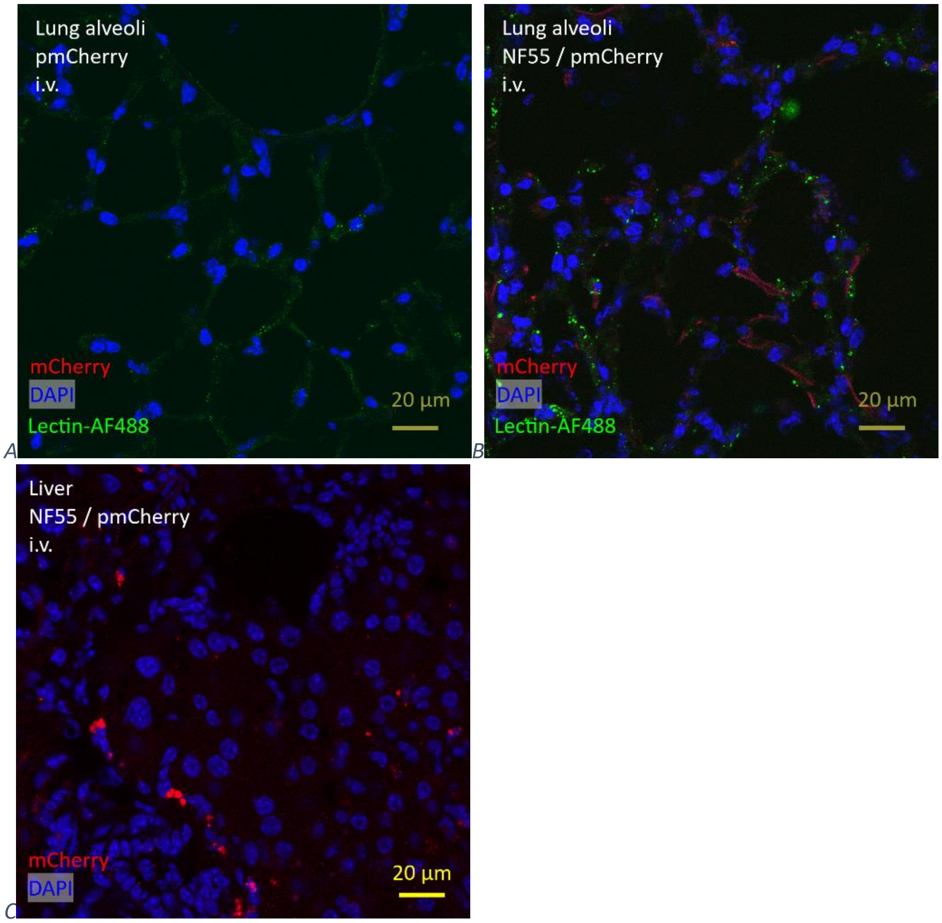

Figure S1. (A) Unvectorized naked pmCherry in lungs. (B) NF55 / pmCherry in Lungs. (C) NF55 / pmCherry in Liver.

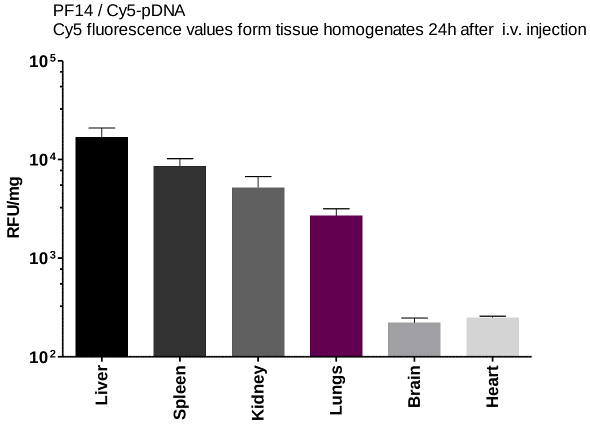

Figure S2. Biodistribution of pDNA after formulation with PF14 at 24h after i.v. injection. The majority of pDNA physically accumulates in liver.

Lung, untreated	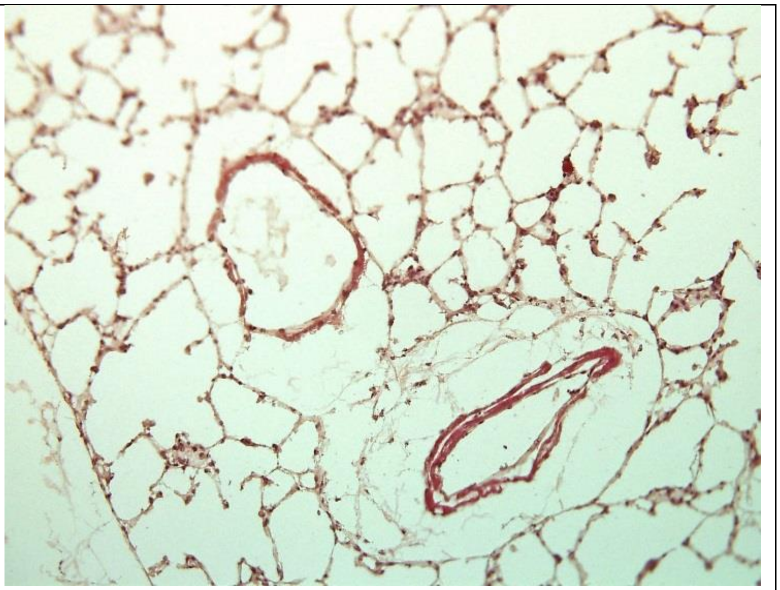
Lung, PF14 / pDNA	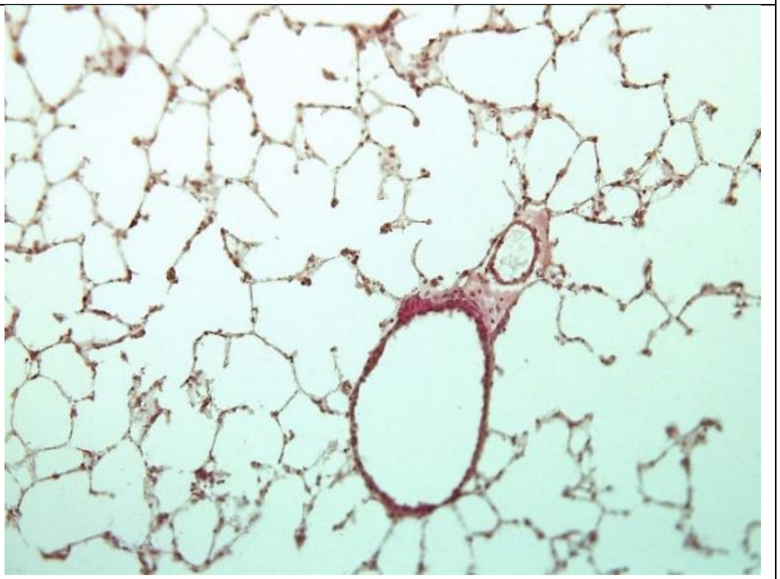

Lung, NF55 / pDNA	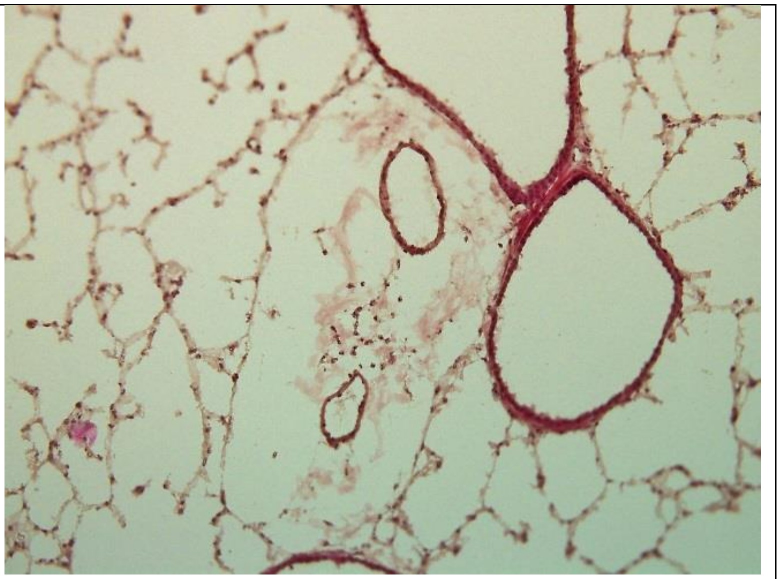

Figure S3. H&E from normal UT mouse and from the animals who were injected with PF14 or NF55 in complex with pDNA.

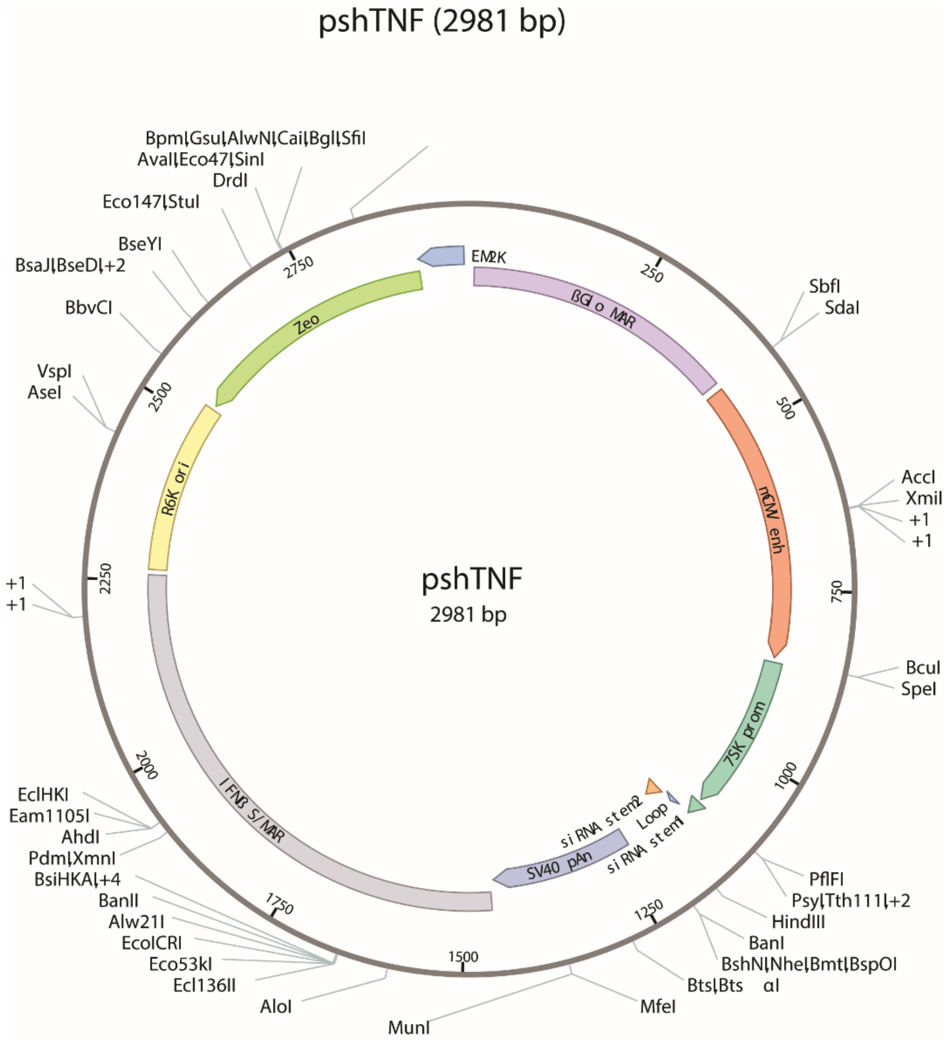

Figure S4. Design of the sh-expressing plasmid: the pshTNF plasmid map.

LPS, Lung, Untreated	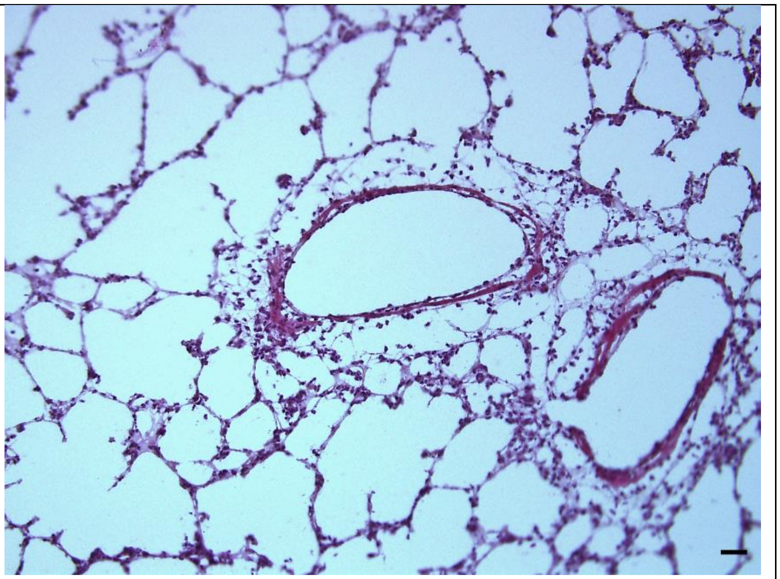
LPS, Lung, siTNF	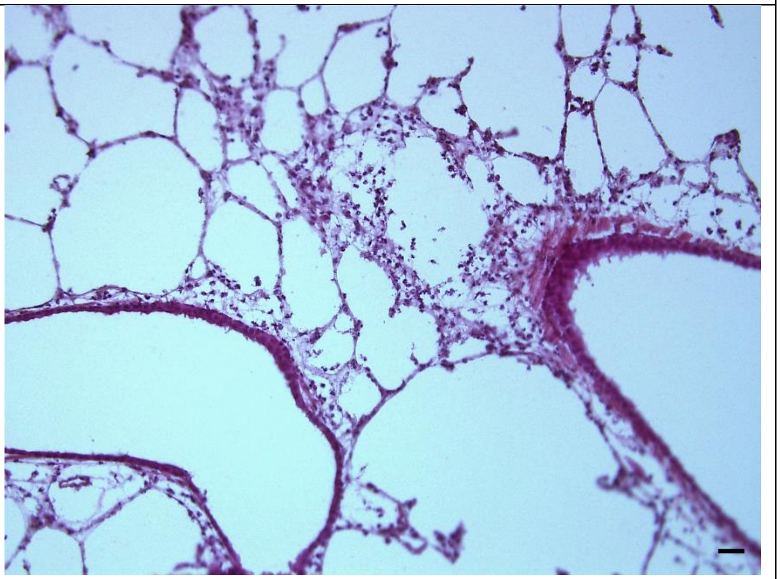

LPS, Lung, PF14 / siTNF	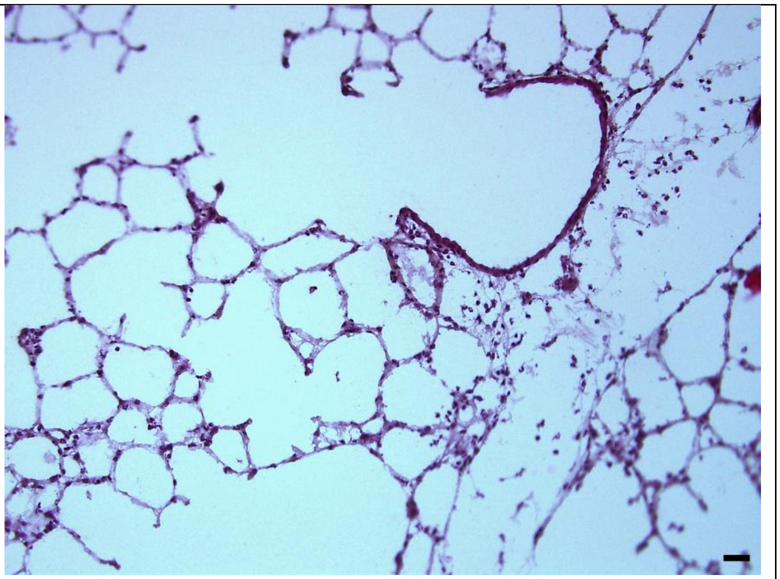
LPS, Lung, NF55 / siTNF	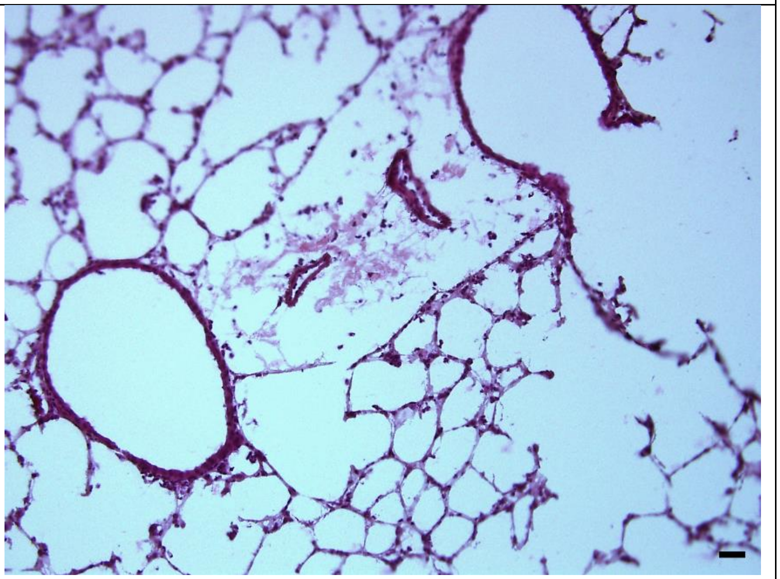

LPS, Lung, NF55 / pshTNF	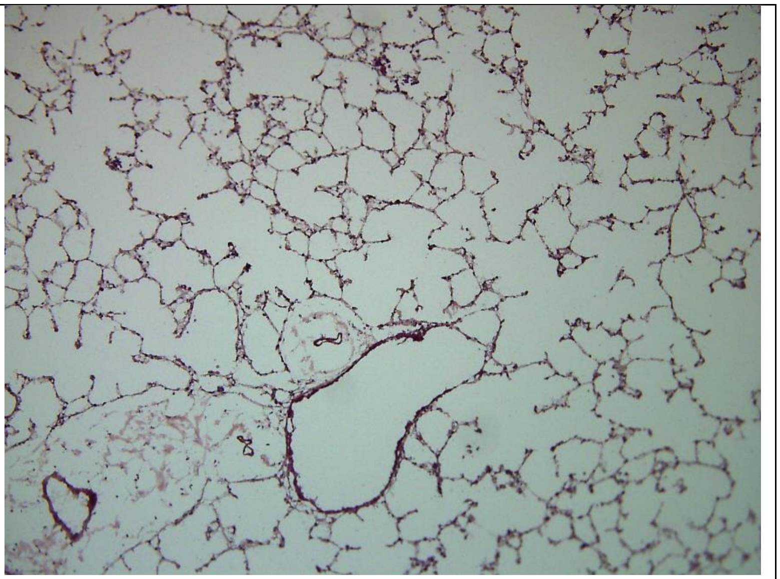
LPS, Lung, PF14 / pshTNF	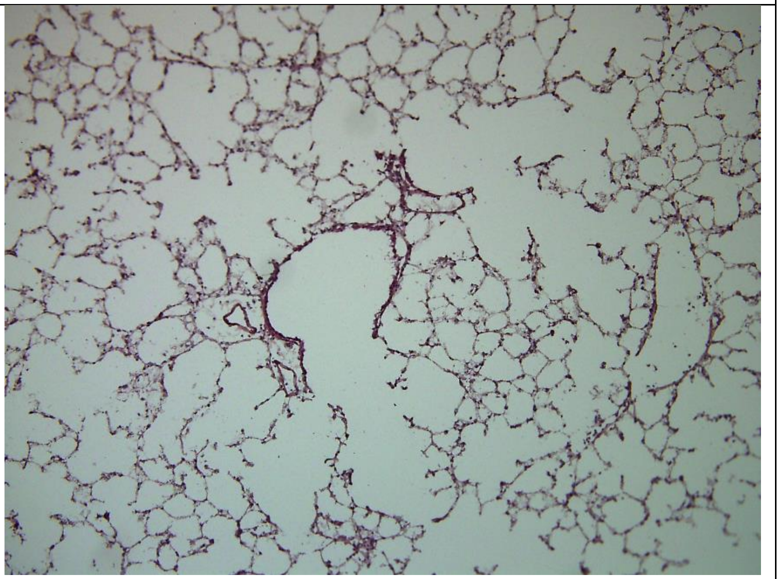

Figure S5. Lung transfection with siTNF or pshTNF inhibits LPS-induced inflammation. H&E stained lung sections of representative individuals. LPS (2.5 mg/kg intranasal) was administered to Balb/C mice at 0h and +24h. siTNF or pshTNF was administered once i.v, at +1h. Lungs were harvested at +28h, cryosectioned, stained with H&E.

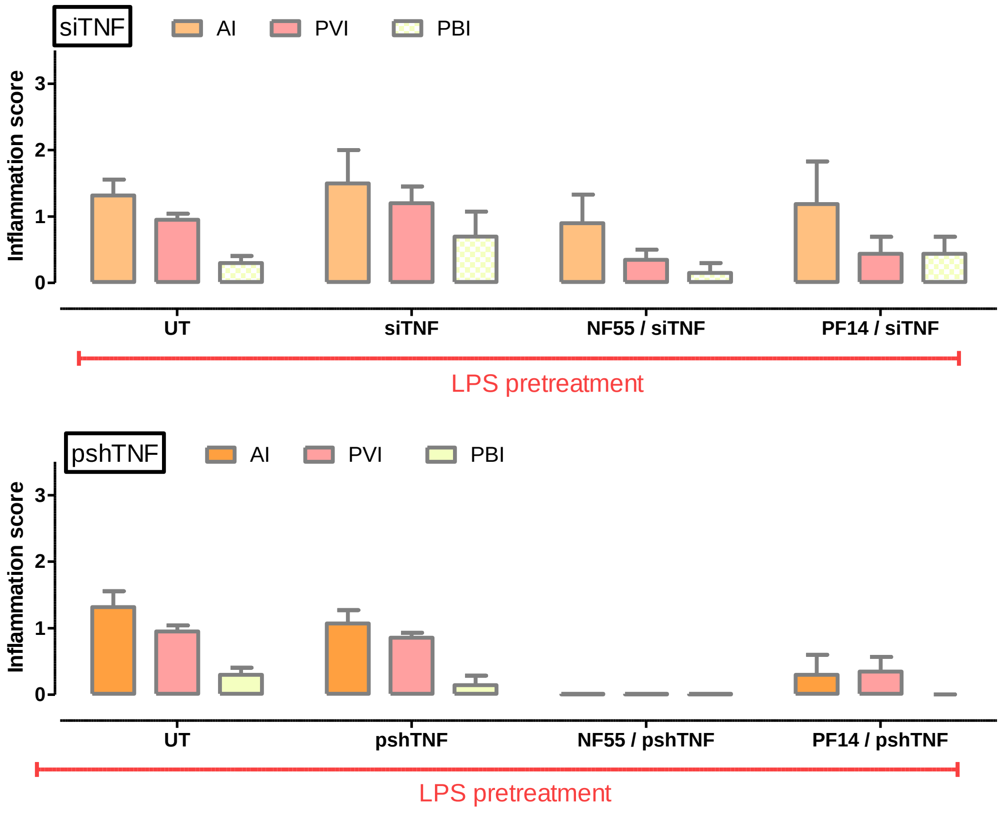

Figure S6. The effect of knocking down TNFα in mice with LPS-induced inflammation by either siTNF or pshTNF. Inflammation was scored by a pathologist: PVI = perivascular infiltration, PBI = peribronchial infiltration, AI = alveolar infiltration.

OVA/ALUM, Lung, pshTNF	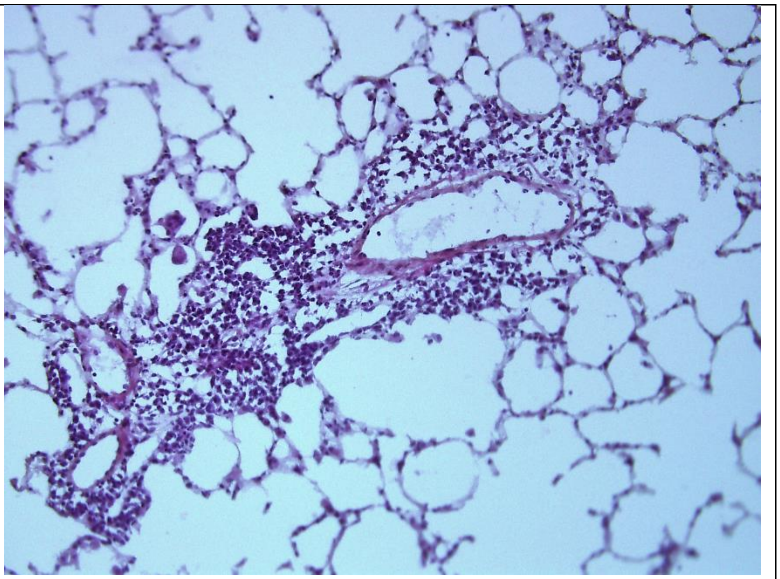
OVA/ALUM, Lung, PF14 / pshTNF	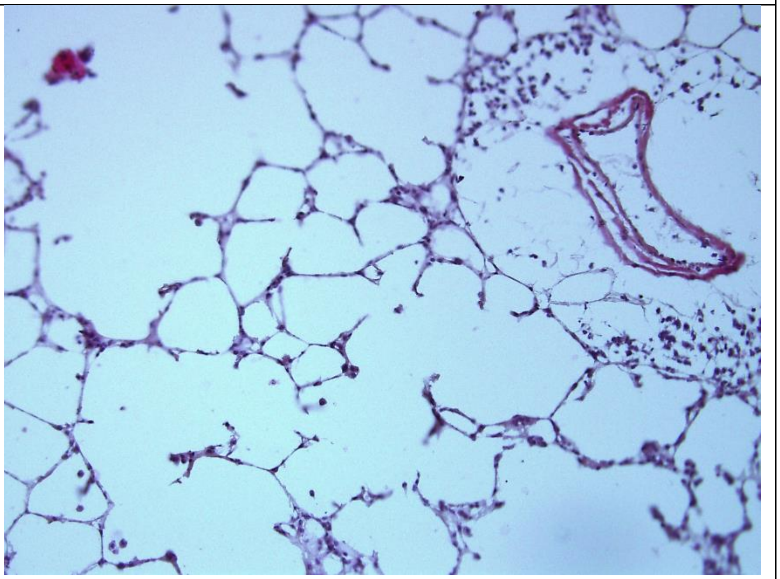

<table>
<tr><td>OVA/ALUM, Lung, NF55 / pshTNF</td><td>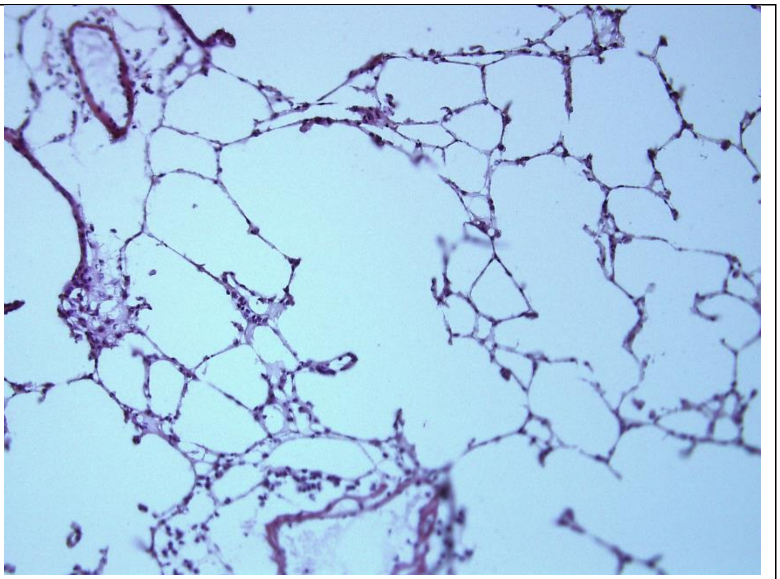</td></tr>
<tr><td>OVA/ALUM, Lung, siTNF</td><td>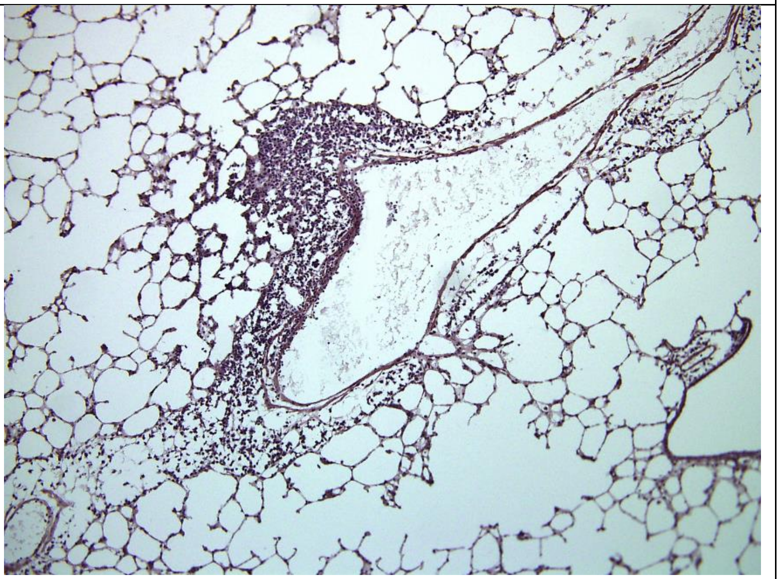</td></tr>
</table>

OVA/ALUM, Lung, PF14 / siTNF	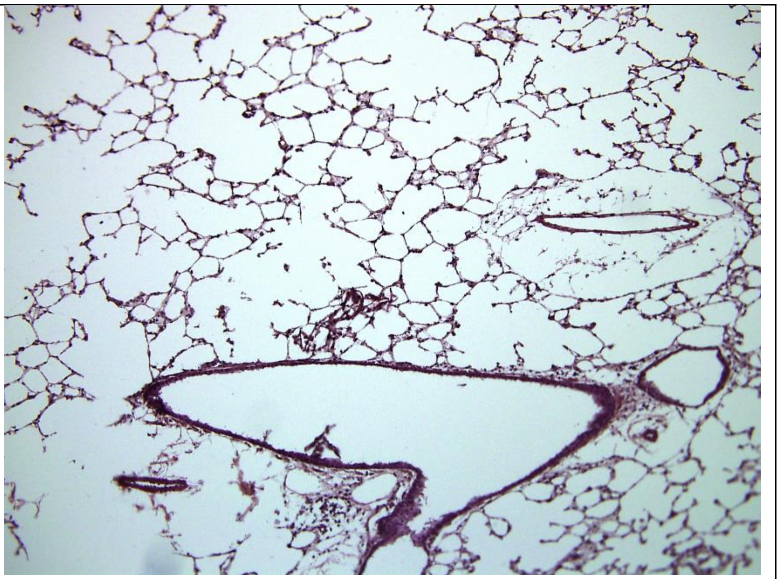
OVA/ALUM, Lung, NF55 / siTNF	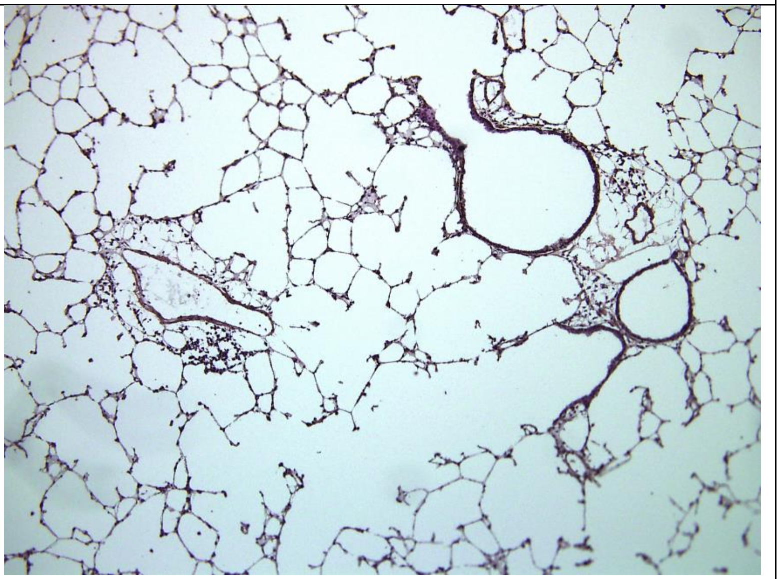

Figure S7. Lung transfection with siTNF or pshTNF inhibits OVA/ALUM-induced inflammation in a model of asthma in mice. H&E stained lung sections of representative individuals.

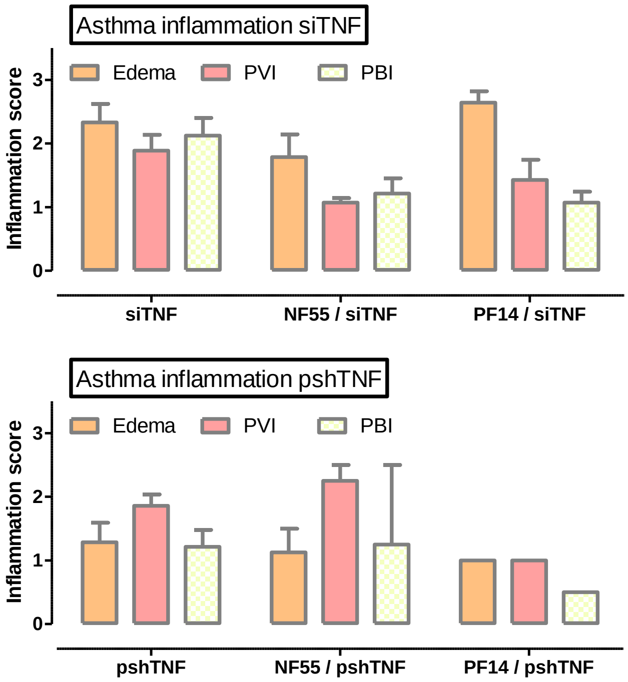

Figure S8. The effect of knocking down TNFα in mice with OVA/ALUM-induced asthma by either siTNF or pshTNF. Inflammation was scored by a pathologist: PVI = perivascular infiltration, PBI = peribronchial infiltration, AI = alveolar infiltration.
